# Remote Americium Detection Using an Optical Sensor: A D-Optimal Strategy for Efficient PLS-Based Modeling

**DOI:** 10.3390/s25227022

**Published:** 2025-11-17

**Authors:** Luke R. Sadergaski, Jeffrey D. Einkauf, Jennifer M. Pyles, Laetitia H. Delmau, Jonathan D. Burns

**Affiliations:** 1 Oak Ridge National Laboratory, Oak Ridge, TN 37830, USA; 2 Department of Chemistry, The University of Alabama at Birmingham, Birmingham, AL 35294, USA

**Keywords:** actinide, americium, chemometrics, experimental design, optical spectroscopy

## Abstract

A fiber-optic visible–near-infrared absorption spectroscopy system in a glove box was demonstrated for remote quantification of Am(III) (0–500 µM) and HNO_3_ (0.1–9 M) using partial least squares regression (PLSR) models. The sensor platform, featuring a simple plug-and-play spectrophotometer, can enable noninvasive, real-time monitoring of actinide process solutions. To establish a flexible PLSR model calibration strategy, a D-optimal design developed using Nd(III) in previous studies was successfully extended to an actinide system with Am(III) to effectively minimize sample set size while maintaining robust prediction performance. The results suggest strong spectral similarities between Nd(III) and Am(III) and validate Nd(III) as an effective optical surrogate for trivalent actinide species. This work also supports the generalizability of a D-optimal training set selection approach for two-factor systems. The PLS1 models for Am(III) and HNO_3_ outperformed a PLS2 model and maintained reasonable performance in the presence of interfering U(VI). The resulting sensor system and multivariate approach provides a flexible and scalable solution for process monitoring, control, and safety in diverse nuclear applications.

## 1. Introduction

Optical sensors play an important role in process analytical technology across diverse sectors, including food production, pharmaceuticals, environmental monitoring, and nuclear technology, in which they support operational efficiency and safety [1,2,3]. Although traditional methods such as inductively coupled plasma mass spectrometry offer superior sensitivity, optical sensors present compelling advantages: they are cost-effective, flexible, nondestructive, and capable of rapid analysis [4,5]. Optical spectroscopy enables real-time, in situ quantification of chemical species, which provides essential online data for process control and optimization. Techniques such as ultraviolet (UV)–visible (vis)–near-infrared (NIR) absorption spectroscopy are particularly well-suited for remote deployment using fiber-optic cables in harsh chemical environments such as radiochemical hot cells [6,7].

Simple univariate analysis (Beer’s law) can be used to quantify molecular or atomic species that absorb light. However, this approach often breaks down in real-world applications when monitoring dynamic processes in which solution conditions alter absorption peaks and molar extinction coefficients [8,9]. Multivariate analysis, or chemometrics, can be used to correlate complex spectral features to analyte concentration in complex systems. Partial least squares regression (PLSR) is a well-established supervised learning method for modeling the relationship between spectral variables (**X**) and analyte concentration (**Y**) in high-dimensional datasets using latent variables (LVs) [10,11,12].

Training supervised multivariate techniques in nuclear field applications can be challenging using one-factor-at-a-time methods because of scarce materials, radiation dose to material handlers, and facility constraints [13,14,15,16]. An optimal design strategy has been proposed to select training set samples for partial least squares (PLS) modeling, with great success in several optical systems (i.e., absorption, fluorescence, and Raman spectroscopy) to minimize sample size while maintaining prediction performance [17,18,19,20,21,22,23,24]. The D-optimal sample selection approach has primarily been validated using lanthanide-based systems (e.g., Nd, Pr), which are assumed to be good spectral surrogates for actinides [25]. However, the electronic structure of lanthanides (4*f*) and actinides (5*f*) results in differences in their absorbance spectra. Actinides tend to have broader and more complex lines, enhanced environmental sensitivity, stronger transition intensity caused by greater orbital mixing, and peaks that occur in the UV–vis and NIR regions [26,27]. These differences are crucial to account for when designing systems for remote sensing and detection of actinides (e.g., Am).

Am is a by-product of nuclear processes, and it is most stable in the +3 oxidation state in HNO_3_ [28,29]. Am has numerous applications, some of which include use in smoke detectors, industrial gauges, isotope production, and as a fuel source for space missions [30,31]. The UV–vis absorption spectrum of Am(III) was first documented many decades ago; however, no work has explored developing multivariate regression models to quantify Am in dynamic conditions for remote process monitoring settings [32,33,34,35]. Separating Am(III) from Cm(III) and trivalent lanthanide elements in most chemical flow sheets is challenging because of their similar chemical properties, and in situ monitoring could help optimize such workflows [36,37].

In this study, fiber-optic cables enabled vis–NIR absorption spectroscopy measurements in a glove box to quantify Am(III) and HNO_3_ using PLSR. Scientific advancement goals in this work were threefold: (1) establish a multivariate approach for the direct quantification of Am(III) (0–500 µM) over a wide range of HNO_3_ concentrations (0.1–9 M), (2) extend a D-optimal design approach to optimize training set samples in an actinide system, and (3) demonstrate a robust framework for rapid, in situ quantification of Am(III) in a glove box using fiber optics and flexible plug-and-play spectrophotometers. The approach can be deployed for remote sensing, even in harsh environments, which makes it valuable for environmental monitoring, chemical processing, and nuclear safeguards applications. Future work should focus on extending the D-optimal training set to include additional factors and expanding the method’s validation across diverse operational scenarios and to further enhance its robustness and applicability.

## 2. Methods and Materials

### 2.1. General Materials

Concentrated HNO_3_ (70%) was purchased from Sigma-Aldrich (Darmstadt, Germany). All samples were prepared using deionized water with a resistivity of 18.2 MΩ·cm and were handled in a negative-pressure glove box. Samples were prepared using calibrated pipettes. The ^241^Am (half-life of 433 years) sample was prepared in-house at the US Department of Energy’s Oak Ridge National Laboratory. The sample was purified from U using an ion exchange column filled with diglycolamide resin. Sample compositions were confirmed by alpha spectroscopy (Canberra Alpha Spectrometer Model 7401, Meriden, CT, USA) and inductively coupled plasma mass spectrometry (iCAP Q, Thermo Fisher Scientific, Waltham, MA, USA). The stock solution contained 2800 µM Am(III).

### 2.2. Absorption Spectroscopy

An Ocean Optics QE Pro high-sensitivity spectrophotometer was used to acquire UV-vis spectra. The spectra were acquired from 315 to 1104 nm with an average spacing of 0.78 nm and 100 ms integration times with a five-scan average. An SL2 Hg/Ar calibration lamp (Stellarnet, Tampa, FL, USA) was used to ensure the instrument was wavelength calibrated. The W–halogen light source (SLS201L, Thorlabs Newton, MA, USA) and QE Pro spectrophotometer were located outside the glove box. Light with output from 360 to 2600 nm was transmitted in and out of a glove box using 400 µm core diameter multimode fiber-optic cables with crush and corrosion resistant stainless-steel jacketing. Sample aliquots were transferred into small-volume cuvettes with a path length of 1 cm. The cuvettes were placed in a cuvette holder for analysis with the lab temperature at 21.5 °C. The spectrophotometer was referenced to deionized water with a resistivity of 18.2 MΩ·cm. Absorption spectra for each sample were collected in triplicate and with cuvette repositioning to estimate repeatability.

### 2.3. Experimental Design

A D-optimal design was generated using Design-Expert (v.22.0.5) by Stat-Ease Inc. (Minneapolis, MN, USA). The design was built using a quadratic process model and contained two continuous factors from zero to one that were chosen using an iterative algorithm that best fit the D-optimality criterion. Factors in this design referred to quantitative variables changed in this experiment [i.e., concentrations of Am(III) and HNO_3_]. The design selected six required model points and six lack-of-fit points for a total of 12 samples based on previous work (Table 1) [23,25]. Lack-of-fit points were included to assess whether the chosen model adequately describes the experimental system. The generic factor levels (i.e., A and B) were scaled to achieve the target concentration levels for each analyte. For example, the Am(III) concentrations were calculated by multiplying factor A concentrations by the maximum concentration in the range (500 µM). This number of samples resulted in a fraction of design space of 0.99. The design is shown in Table 1, and the resulting spectra are shown in Figure S1. The validation set was generated using a one-factor-at-a-time approach with four Am(III) and eight HNO_3_ concentration levels (40 samples).

### 2.4. Chemometrics

The Unscrambler X (version 10.4) software packaged by CAMO Analytics was used for PLSR modeling and preprocessing. Savitzky–Golay (SG) smoothing and first derivatives with varying polynomial orders and smoothing points were applied to the data to help remove noise artifacts. Replicate spectra were averaged for each calibration sample to build PLSR models. A baseline offset was calculated by subtracting the lowest points in the spectrum from all the variables. Spectra were also trimmed to include only the peaks of interest or larger regions of the spectrum. Spectra were mean-centered, and a nonlinear iterative partial least squares (NIPALS) algorithm was used for PLSR [10]. PLS2 and PLS1 models were created for Am and HNO_3_ concentrations. PLS1 models one Y variable and PLS2 simultaneously models more than one Y variable. The number of LVs in PLSR models was chosen by the last significant decrease (>10%) in the root mean square error (RMSE) of cross validation (RMSECV) statistics using a full leave-one-out approach.

A pseudounivariate limit of detection (*LOD*_pseudo_) for PLSR models was calculated using Equation (1):

(1) LODpsuedo=3.3spseudo−11+h0min+1nvarpsuedo

where *s*_pseudo_ is the slope of the known calibration sample concentrations plotted against the model-predicted calibration sample concentrations, *h*_0min_ is the minimal calibration sample leverage, *n* is the number of calibration samples, and *var*_pseudo_ is the variance of the model-predicted calibration sample concentrations [38]. These *LOD*_pseudo_ values are estimates and can be either similar or conservative when compared with more calculation-involved limit of detection (LOD) confidence bands [39].

### 2.5. E: Statistical Comparison

PLSR performance was evaluated using calibration, cross validation, and prediction statistics. A balanced model generally has similar RMSEs of calibration (RMSEC), cross validation (RMSECV), and prediction (RMSEP). The predictive power was evaluated using RMSEP, percent RMSEP (RMSEP%), bias, and standard error of prediction (SEP). RMSEs were calculated using Equation (2):

(2) $$RMSE=\sqrt{\frac{\sum_{i=1}^{n}{\left({\hat{y}}_{i}-y_{i}\right)}^{2}}{n}}$$

where ${\hat{y}}_{i}$ is the predicted concentration, *y_i_* is the measured concentration, and *n* is the number of samples. The RMSE% was calculated by dividing the RMSE by the midpoint of the **Y** matrix concentration range using Equation (3):

(3) $$RMSE\%=\frac{RMSE}{\bar{y}}\times 100\%$$

where $\bar{y}$ is the mid-point value. RMSE values are reported in analyte units. Bias values close to zero are important when using minimized training sets. Values of RMSE% ≤ 10% indicate acceptable performance, and values of RMSE% ≤ 5% indicate strong performance [23].

## 3. Results and Discussion

### 3.1. Absorption Spectra

**Figure 1:**
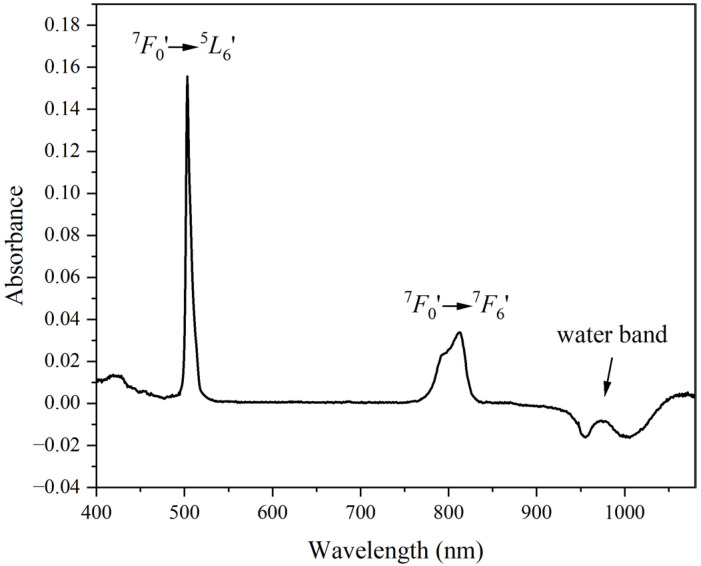
Absorption spectra of a solution containing 500 µM Am(III) in 3 M HNO_3_, with labeled peaks.

The absorption spectrum of Am(III) in various media has been established primarily using stationary benchtop spectrophotometers [34,35]. In the vis–NIR region, the Am(III) absorption spectrum is dominated by a peak near 503 nm and another peak near 810 nm. The sharp absorption peak near 503 nm and the broad peak near 810 nm correspond to the ^7^*F*_0_′–^5^*L*_6_′ and ^7^*F*_0_′–^7^*F*_6_′ electronic transitions, respectively (Figure 1) [35]. In addition to Am(III) spectral features, the NIR water band near 960 nm is evident (Figure 1). This NIR band is much less intense than water bands in longer-wavelength regions [18]. Two dips were noted near 957 and 1007 nm, which are consistent with previous findings [40]. These features correspond to a combination band involving the O–H stretching and bending vibrations of water. The HNO_3_ matrix perturbs the H-bonding network of water, resulting in a peak that increases in intensity with increasing HNO_3_ concentration (Figure S2). The water band is also sensitive to ionic strength and temperature, but the concentration of Am(III) in this study was too low to affect water band features.

**Figure 2:**
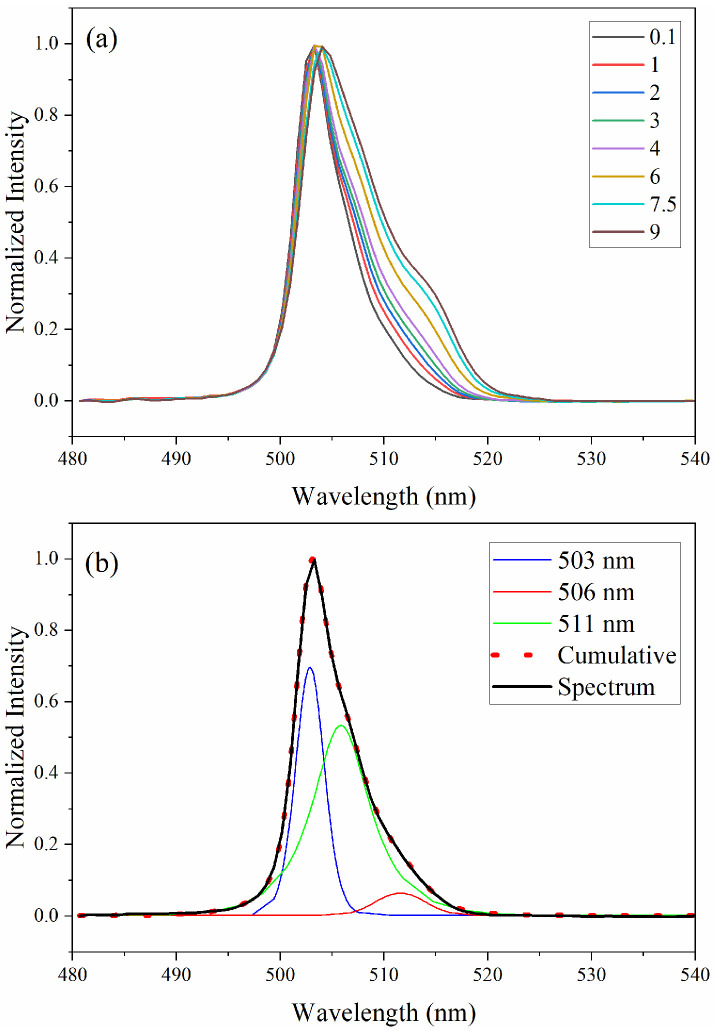
Example absorption spectra (**a**) normalized 503 nm peak samples with 500 µM Am(III) in 0.1–9 M HNO_3_ and (**b**) peak fit analysis of a sample with 500 µM Am(III) in 1 M HNO_3_.

Ligands have a strong effect on the inner f–f orbital transitions of Am(III). The absorption bands are sensitive to HNO_3_ concentrations because of Am(III) complexation with nitrate (NO_3_^−^) [28,34,35]. The absorption band near 503 nm systematically decreases in intensity with increasing HNO_3_ concentration. The peak height normalized spectra are shown in Figure 2 to emphasize the changing shape and redshift of this band with increasing HNO_3_. The 503 nm Am(III) peak shape is asymmetrical and appears to have multiple shoulders (Figure 2).

Gaussian–Lorentzian peak fitting of the 503 nm band reveals multiple peaks located near 503, 506, and 511 nm (Figure 2b). The peak is likely a superposition of multiple transitions corresponding to closely spaced energy levels. Each energy level corresponds to transitions between different *J*-levels (total angular momentum) of the ground and excited configurations [35]. Solvent interactions and ligand field effects smear the fine structure in the band, making each level challenging to resolve. However, the plug-and-play spectrometer has sufficient resolution to partially resolve the peaks. A first derivative could be applied to enhance resolution by highlighting inflection points. Changes in the contribution from the three peaks result in an apparent redshift and spectral shape changes with increasing HNO_3_. For example, the 503 nm peak contribution decreases in intensity, but the 511 nm peak increases and redshifts slightly to 513 nm at 9 M HNO_3_. The intensity of the fitted peak of reach HNO_3_ concentration is shown in Table S1.

### 3.2. Limits of Detection

The LOD is defined as the lowest concentration that can be detected with reasonable certainty for a given method. The LOD and limit of quantification (LOQ) were estimated by univariate models for different HNO_3_ levels. The LOD and LOQ were calculated using Equations (4) and (5):

(4) $$LOD=\frac{3\times s}{m}$$

(5) $$LOQ=\frac{10\times s}{m}$$

The variable *s* is the standard deviation or noise of the blank, and *m* is the slope obtained by plotting the intensity of the peak versus concentration. An example linear calibration curve is shown in Figure S3. The coefficient of determination (*R*^2^) for each linear curve is between 0.9980 and 0.9999. The calculated LOD, LOQ, slope, *R*^2^ values, and molar extinction coefficients for peak maxima are shown in Table 2. The 503 nm peak intensity gradually decreases with increasing HNO_3_, which results in less sensitivity with higher HNO_3_.

The absorption spectra were collected near room temperature (22 °C) and with a 1 cm pathlength in this experiment with a plug-and-play spectrophotometer. Molar extinction coefficients can vary based on instruments and instrument parameters such as spectral band width or resolution. Changes in sample temperature or the pathlength would significantly affect LODs. Given the spectral changes as a function of HNO_3_, a univariate approach for quantification is not practical unless the given concentrations of HNO_3_ are known. In processing applications, Am(III) and HNO_3_ levels would be unknown and continuous due to mixing gradients [11,13]. Therefore, a multivariate approach for the direct quantification of Am(III), without a priori knowledge of solution conditions, was evaluated.

### 3.3. D-Optimal Design

D-optimal designs are well-suited for constructing efficient and informative training data. The prediction performance of supervised PLSR models is highly dependent on the number and type of samples in the training set. With too many samples, variance dominates the error, and with too few samples, bias will dominate [15]. Recent studies of numerous systems have shown that D-optimal designs can reduce training set size while maintaining or even improving PLSR performance [22,24,25]. Unlike classical factorial designs, which can be large and rigid, D-optimal designs use a mathematical criterion—maximizing the determinant of the information matrix—to select the most important samples within a given factor space. When employing full factorial designs, it is common to use 5 levels for each factor, resulting in 25 calibration samples (5^2^) [14]. However, a D-optimal approach yielding only 12 samples, represents a sample reduction of more than 50% [6]. D-optimal designs can help mitigate overfitting, improve generalizability, and can be adapted to constraints such as viable experimental conditions to capture the anticipated variation.

D-optimal designs are highly practical for real-world applications in which time, cost, and sample availability are constrained. Most applications have not extended the D-optimal approach to an actinide-based spectral system but have demonstrated feasibility using lanthanides [25]. The ability for a D-optimal design to adequately sample the relevant chemical and spectral variation in an actinide system [i.e., Am(III)] must be determined experimentally. Therefore, a D-optimal design based on a quadratic model was evaluated in this work, as previously established using absorption spectroscopy for lanthanides (e.g., Nd) and Raman spectroscopy for U(VI), identifying approximately 12 samples as optimal [25,41]. The variation in the hypersensitive Nd(III) absorption band was assumed to be a reasonable spectral surrogate for actinides such as Am(III) [9]. This assumption was evaluated in this work to determine whether the design space coverage could adequately capture the spectral variation in complex Am(III) absorption spectra. Further establishing the generalized D-optimal design in this diverse actinide system could provide additional confidence in the approach and allow it to be seamlessly leveraged by researchers studying comparable two-factor systems. D-optimal designs are not limited to two-factor systems; they can be extended to incorporate variables such as temperature or additional analytes [20,23]. Most modern statistical software options (e.g., Design-Expert by Stat-Ease Inc.) can handle a moderate number of factors (10–20). Extending the D-optimal approach to systems with more than two factors is expected to yield even greater improvements in training set efficiency compared to traditional full factorial models [25].

### 3.4. Partial Least Squares Regression

Am(III) absorption spectra contain overlapping and colinear features that are difficult to capture using univariate methods. PLSR is well-suited for capturing subtle shifts in Am(III) absorption peaks and water bands for quantitative analysis. Even when peak shifts are not fully resolved, PLS can identify relationships between spectral shape changes and concentration. One advantage of using PLSR over univariate models is that it can accurately predict Am(III) without needing prior information about sample acidity [23]. A PLS2 model was compared with PLS1 models to see if simultaneously modeling both response variables provided an advantage. PLS2 models more than one **Y** variable at a time and can provide benefits when analytes are chemically or spectrally correlated and can reduce computational burden and maintenance in industrial or real-time applications. However, PLS2 can be more sensitive to noise and multicollinearity. In this study, each PLSR model was generated using the D-optimal training set (12 samples).

PLS1 models for Am(III) and HNO_3_ were built using only the 503 nm Am(III) peak or the water band region from 900 to 1050 nm and were then compared with PLS2 models that evaluated both peaks and analytes simultaneously. NIR water bands can be used to determine acid concentration, even for acids that do not directly absorb NIR because acid perturbs the vibrational structure of water [18]. Leveraging the water band for HNO_3_ predictions has the advantage of being able to estimate HNO_3_ levels even when Am(III) is not present in solution, which is particularly advantageous for monitoring applications [23].

**Figure 3:**
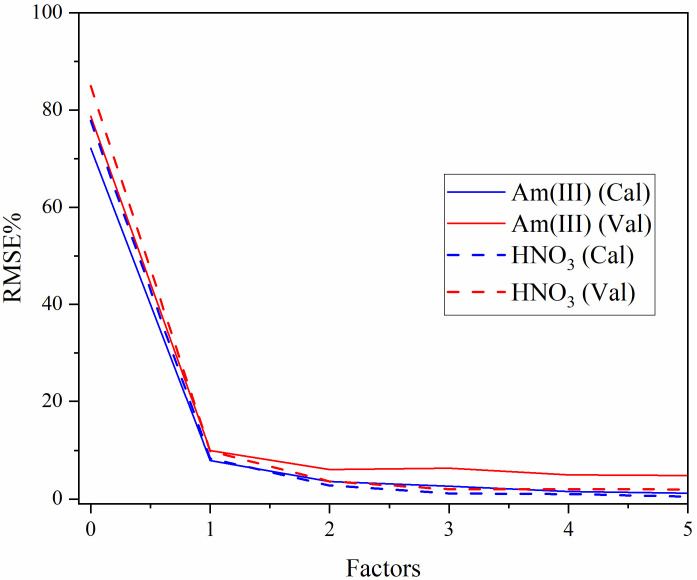
Plot of RMSE% versus the number of LVs for Am(III) and HNO_3_.

The last significant decrease in RMSECV was used to determine the optimal number of LVs in each model. The RMSE% values for Am(III) and HNO_3_ PLS1 models are shown in Figure 3. The optimal trade-off between model complexity and predictive accuracy was three LVs for the HNO_3_ model and two LVs for Am(III) because the RMSECV was no longer reduced after these points. This number of LVs is reasonable considering the complexity of the spectral features caused by changes in speciation and the number of species present [e.g., HNO_3_, H^+^, NO_3_^−^, Am(III), AmNO_3_^2+^]. Additional components past three LVs are likely modeling noise and would result in overfitting.

A summary of calibration, cross validation (CV), and prediction metrics is shown in Table 3. The explained **Y**-variance was greater than 99% for each PLSR model. The PLS2 model required two LVs and resulted in RMSEP% values of 4.0% and 9.2% for Am(III) and HNO_3_, respectively. The PLS1 model for Am(III) slightly outperformed the PLS2 model, with an RMSEP% value of 3.5%. The 503 nm Am(III) peak was smoothed using an SG filter with a third-order polynomial and seven smoothing points and was then corrected using a baseline offset. A third-order polynomial is commonly used to model subtle variations in spectral features, which can be important if the absorption bands have asymmetries or more complex shapes. The RMSEP value of 8.7 is an estimate of the plus or minus error in the predictions and is reasonable considering the estimated univariate LODs shown in Table 2, which are around 8–12 µM. The *LOD*_pseudo_ for Am(III) is 29 µM, which is greater than even two times the RMSEP—a relatively conservative estimate of the error. This result is consistent with previous findings that suggest *LOD*_pseudo_ can be either consistent with or conservative compared to the true limit of detection [39]. The PLSR *LOD*_pseudo_ value for Am(III) is likely higher compared to the univariate models (Table 2) because the PLSR model accounted for spectral variability due to different HNO_3_ levels while the univariate models did not. The average percent relative standard deviation calculated for the Am(III) validation sample replicates was less than 1%, demonstrating strong repeatability.

The RMSEP% value for HNO_3_ decreased from 9.2% to 3.8% when using a PLS1 model. SG filters are useful for smoothing spectra and correcting baseline offsets while maintaining shape. Thus, an SG derivative with a first-order polynomial and 15 smoothing points, which is better tailored to the broad NIR features, was used for the PLS1 HNO_3_ model. An SG derivative with a first-order polynomial and nine smoothing points was used for the PLS2 model to balance the sharp Am(III) features with the broad NIR features. However, derivatives can suppress broad bands relative to sharp peaks. This suppression likely occurred in the PLS2 model, which enhanced the Am(III) peak relative to the NIR water band.

A reasonable difference of approximately twofold or less between RMSEC and RMSECV indicates that the D-optimal training set was representative of the variation in expected future samples and that the model did not overfit the calibration data. The somewhat elevated RMSECV values compared with RMSEC values for Am(III) were expected with a minimized sample training set, and this result suggests that performance would diminish with fewer than 12 training samples. Low bias values indicate that the models did not consistently over- or underestimate the true values. Balanced RMSEC and RMSECV values with low bias suggest that despite the limited spectra in the training set, PLSR still captures the underlying relationships between predictors and response.

The RMSEP values were similar to the RMSEC values for each model shown in Table 3. For Am(III), RMSEP% values for PLS2 and PLS1 models were below 5%, which indicates strong performance [23]. An RMSEP% value of 3.8% for HNO_3_ is also considered strong performance and can provide valuable estimates of HNO_3_ concentration in process solutions with and without the presence of Am(III). Estimates of HNO_3_ concentration can be modeled using the Am(III) peak directly; however, this model requires that Am(III) is present in solution, so it is less advantageous for process monitoring.

**Figure 4:**
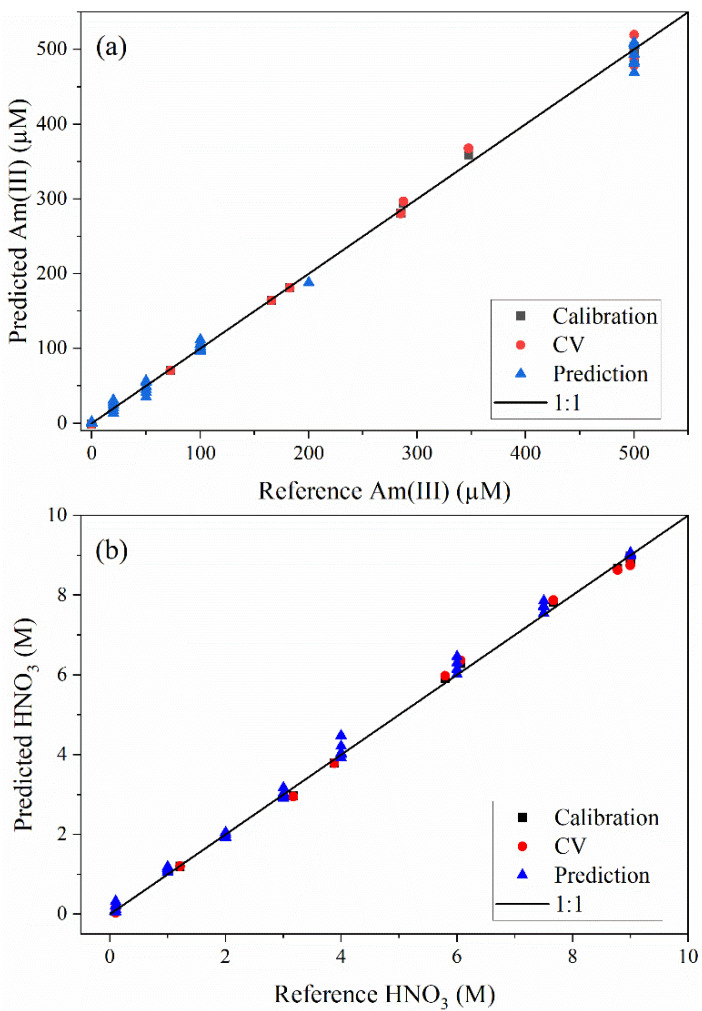
Parity plots for (**a**) calibration, CV, and prediction metrics for PLS1 model built for Am(III) and (**b**) calibration, CV, and prediction metrics for PLS1 model for HNO_3_.

Parity plots summarizing the calibration, CV, and prediction performance for PLS1 models are shown in Figure 4. The calibration set contained 12 samples, and the validation set contained 40 samples. The CV samples are near the 1:1 line, indicating that leaving one sample out did not significantly inflate the RMSE. The estimated sample concentrations in the external validation set are also close to the 1:1 line. This result indicates robust prediction performance over the entire range for both Am(III) and HNO_3_.

### 3.5. Performance in the Presence of U

PLSR models evaluate spectral profiles rather than univariate methods that rely on isolated wavelengths. Multivariate analysis can outperform univariate methods when interferences are present [8]. Generating pure Am(III) solutions is challenging, and Am(III) is often accompanied by impurities such as lanthanides or other actinides like Cm(III) or U [36,37]. Therefore, the PLS1 model performance in the presence of U(VI) was evaluated to determine how well the model can perform in the presence of an interferent and to evaluate diagnostic capabilities typical of an overdetermined system.

**Figure 5:**
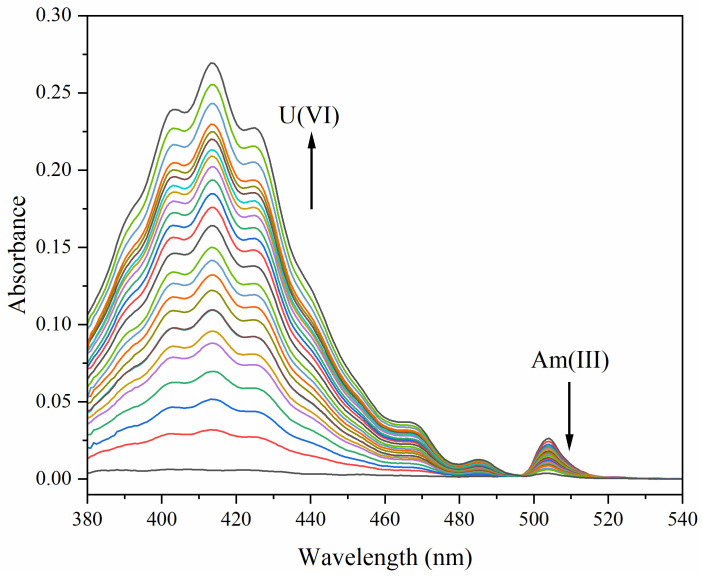
Absorption spectra of a 100 µM Am(III) solution in 1 M HNO_3_ with incremental dilutions (16 µL aliquots) of a U(VI) stock solution. The last three samples had larger 32 µL aliquots.

A 100 µM Am(III) sample in 1 M HNO_3_ was gradually diluted with a U(VI) stock solution. At each increment, the concentration of U(VI) increased, the concentration of Am(III) decreased while HNO_3_ was kept constant, and a spectrum was recorded (Figure 5). The final U concentration was 40 mM compared with just 30 µM Am(III).

A Hotelling’s T^2^ statistic and Q-residual values for the Am(III) predictions were evaluated based on an F-test (*p*-value of 1%). These can be useful for identifying outliers or off-normal situations and can be visualized simultaneously using a Influence plot. The Hotelling’s T^2^ distance measures how far a new sample projection is from the center of the multivariate space described by a PLSR model. Q-residuals measure the amount of variance in a sample that is not explained by the model. If a sample has high Q-residuals, then the spectrum likely contains unusual features not captured by the PLSR model. This metric can be used to flag samples that are poorly modeled because of noise, errors, or fundamental differences (e.g., interferences).

**Figure 6:**
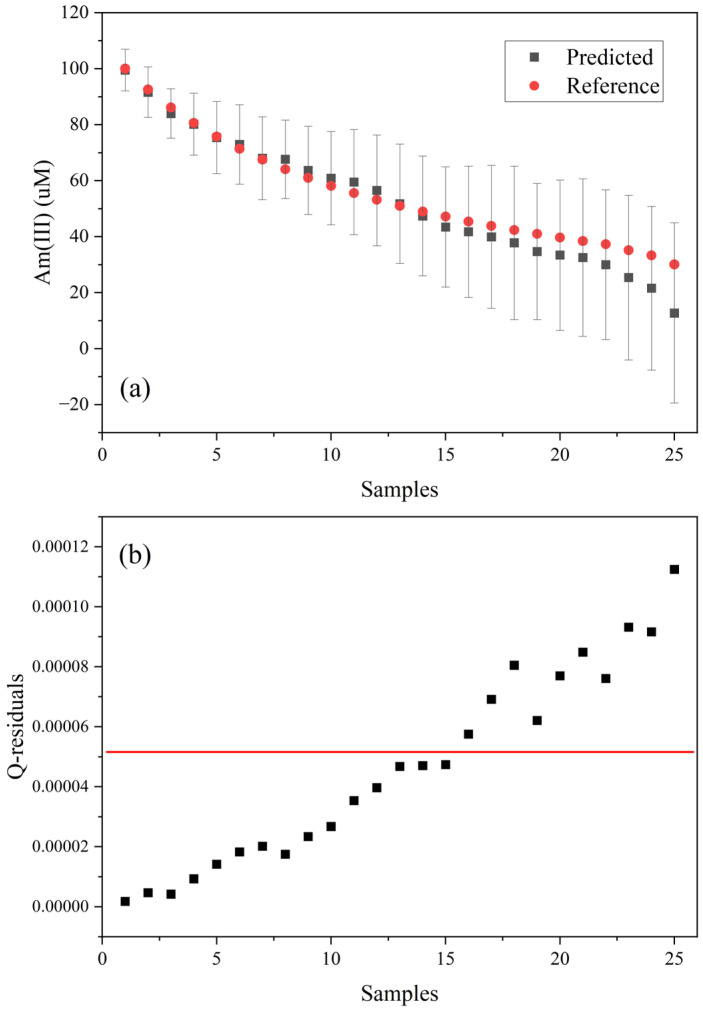
Plot of (**a**) predicted Am(III) concentration against reference values and (**b**) Q-residuals with 1% critical limit (red line).

Figure 6 shows the PLS1 model predicted and reference Am(III) concentration values for each spectrum, along with the Q-residuals. After the ratio of 45 µM Am(III) and 26 mM U, the Q-residuals become large, and the estimated deviation in the predictions begin to inflate significantly. The PLS1 model outperformed the PLS2 model in this scenario. The PLS2 model was built using a larger portion of the spectrum from 460 to 1080 nm. The Q-residuals became inflated after the first addition of U(VI), which is beneficial for fault detection, but the Am(III) predictions were not as accurate (Figure S4). Tailoring PLS1 models to a narrow region of the spectrum in this scenario had the advantage of maintaining prediction performance even in the presence of an interferent.

The PLS1 approach provided robust Am(III) concentration estimates with varying HNO_3_ levels, even in the presence of low levels of U(VI) interferences. To better account for multi-actinide systems and greater levels of spectral interferences, the training set should include mixed samples measurements. This could be accomplished using a D-optimal design with additional factors [20]. The 503 nm Am(III) peak provided great sensitivity down to single micromolar concentration levels in this study. However, the less intense Am(III) peak near 810 nm was not modeled in this work. The lower absorptivity of this peak could be leveraged in combination with the 503 nm Am(III) peak in a future study to expand the measurable Am(III) concentration range, much like a hierarchical PLSR model developed in a recent study to cover a wide range of Np(V) concentrations [42]. It should also be noted, that the current study was performed under controlled conditions, and performance may vary when applied over time in even more complex or harsh environments where radiation damage could impact the long-term performance of optical elements [43]. Future work will focus on expanding the method’s validation across diverse operational scenarios and calibration transfer strategies to further enhance its long-term applicability [44].

## 4. Conclusions

This study demonstrated the effectiveness of PLSR for the direct quantification of Am(III) (0–500 µM) and HNO_3_ (0.1–9 M) using vis–NIR absorption spectroscopy via fiber-optic cables in a glove box. The Am(III) absorption peak near 503 nm was sensitive to HNO_3_ concentration, and the changes were likely a result of nitrate complexation. The variance in the Am(III) spectral features was captured by a training set selected using D-optimal design. The designed approach, which was originally developed with lanthanides in previous studies, effectively minimized training set samples while maintaining strong prediction performance for an actinide system containing Am(III). This result suggests that lanthanides such as Nd(III) are useful spectral surrogates for actinides and that the generalized D-optimal design can be applied with confidence to diverse chemical systems. PLS1 models tailored to specific regions for Am(III) and HNO_3_ outperformed a PLS2 model that used the entire spectrum. The PLS1 model also maintained reasonable performance for Am(III) quantification in the presence of U(VI) interferences. The system can enable real-time monitoring of solutions containing Am(III) and HNO_3_ to support diverse applications such as radiochemical separations.

## Figures and Tables

**Table 1:** D-optimal design sample types and target concentrations of Am(III) and HNO_3_.

Sample	A	B	Am(III) (µM)	HNO_3_ (M)	Space Type	Build Type
1	0.000	1.000	0.0	9.0	Vertex	Model
2	1.000	0.850	500.0	7.7	Vertex	Model
3	0.200	0.345	100.0	3.2	Interior	Model
4	0.365	1.000	182.5	9.0	Edge	Model
5	0.331	0.670	165.7	6.1	Interior	Lack of fit
6	0.000	0.640	0.0	5.8	Interior	Lack of fit
7	1.000	0.000	500.0	0.1	CentEdge	Lack of fit
8	1.000	0.425	500.0	3.9	Edge	Lack of fit
9	0.695	0.975	347.5	8.8	Edge	Model
10	0.575	0.000	287.5	0.1	Vertex	Model
11	0.145	0.000	72.5	0.1	Interior	Lack of fit
12	0.570	0.125	285.0	1.2	Interior	Lack of fit

**Table 2:** LOD and LOQ values for Am(III) at different HNO_3_ concentrations.

HNO_3_ (M)	LOD (µM)	LOQ (µM)	Peak Maximum (nm)	Slope (m)	Extinction Coefficient (cm^−1^ M^−1^)
0.1	2.4	8	503.3	3.28 × 10^−4^	327
1	2.5	8.3	503.3	3.19 × 10^−4^	319
2	2.5	8.4	503.3	3.13 × 10^−4^	314
3	2.6	8.7	503.3	3.03 × 10^−4^	302
4	2.7	9.1	503.3	2.88 × 10^−4^	290
6	3.2	10.7	503.3	2.46 × 10^−4^	243
7.5	3.6	11.9	504.0	2.21 × 10^−4^	221
9	3.7	12.2	504.0	2.15 × 10^−4^	216

**Table 3:** Summary of PLSR model calibration and validation metrics.

Variables	PLS2	PLS1 Am(III)	PLS1 HNO_3_
LVs	3	2	3
Region (nm)	460–1080	480–530	900–1080
Preprocessing	First derivative	Smooth/baseline offset	First derivative
Am(III)	—	—	—
RMSEC	10	5.4	—
RMSECV	18	10	—
RMSEP	9.9	0.87	—
RMSEP%	4.0	3.5	—
SEP	10	9.3	—
Bias	−0.17	0.86	—
HNO_3_	—	—	—
RMSEC	0.32	—	0.12
RMSECV	0.52	—	0.16
RMSEP	0.41	—	0.17
RMSEP%	9.2	—	3.8
SEP	0.37	—	0.15
Bias	0.18	—	0.097

## Data Availability

The original contributions presented in this study are included in the article/Supplementary Material. Further inquiries can be directed to the corresponding authors.

## Supplementary Materials

The following supporting information can be downloaded at: https://www.mdpi.com/article/10.3390/s25227022/s1, Figure S1: D-optimal training set of Am(III) absorption spectra with baseline offset; Figure S2: Near-infrared (NIR) water band region with calibration (12) and validation (40) spectra after applying a first derivative. Arrows indicate the direction of intensity increases with increasing HNO_3_ concentration; Figure S3: Linear regression of peak intensity vs. Am(III) concentration for samples collected at 4 M HNO_3_. Figure S4: Q-residuals for predicted Am(III) samples while diluting with U(VI) using the PLS2 model. The red horizontal line represents the critical limit (1%). Table S1: Gaussian–Lorentzian peak fit intensities for normalized peaks near 503 (1), 506 (2), and 511 nm (3). Peaks were fitted with OriginPro software.
