# Supplementary material for: Remote Americium Detection Using an Optical Sensor: A D-Optimal Strategy for Efficient PLS-Based Modeling
Source: Sensors (Basel). 2025 Nov 17;25(22):7022. doi: 10.3390/s25227022 (PMC12656064; doi:10.3390/s25227022)
Supplement: Supplementary file 1 [file sensors-25-07022-s001.zip › sensors-3972069-supplementary.pdf]

This document contains the following:

Tables: 1

Figures: 4

Pages: 4

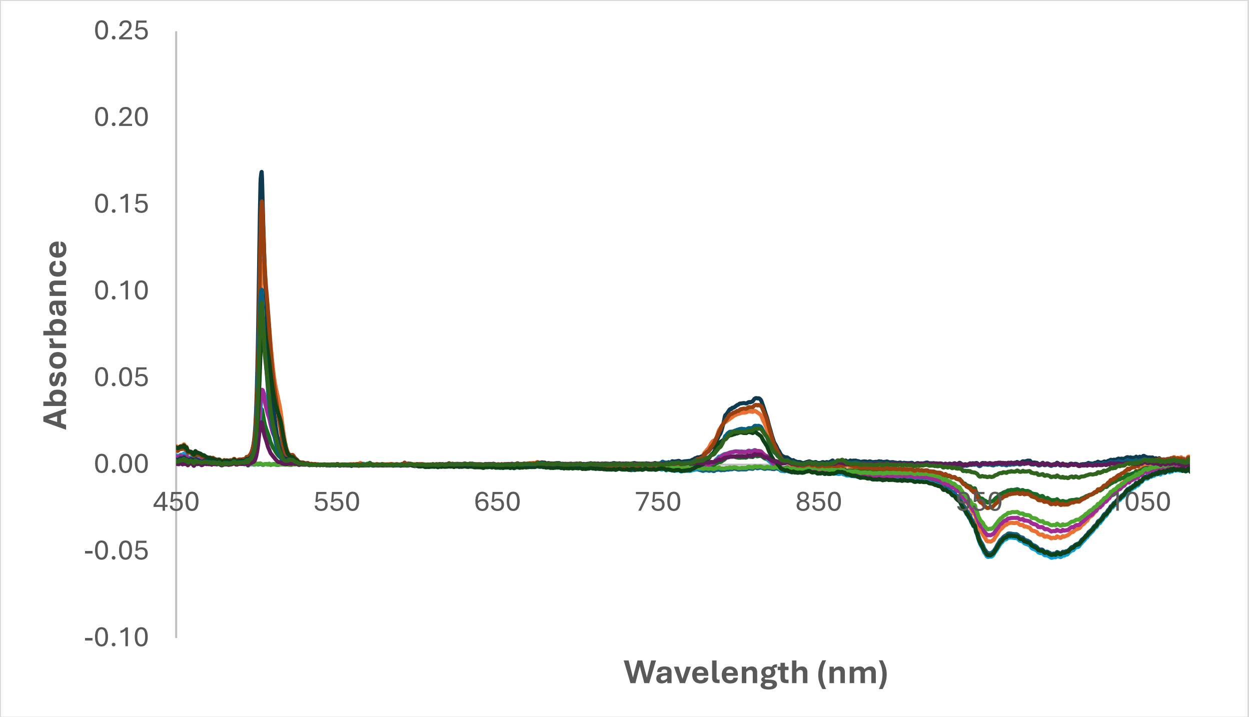

Figure. S1. D-optimal training set of Am(III) absorption spectra with baseline offset.

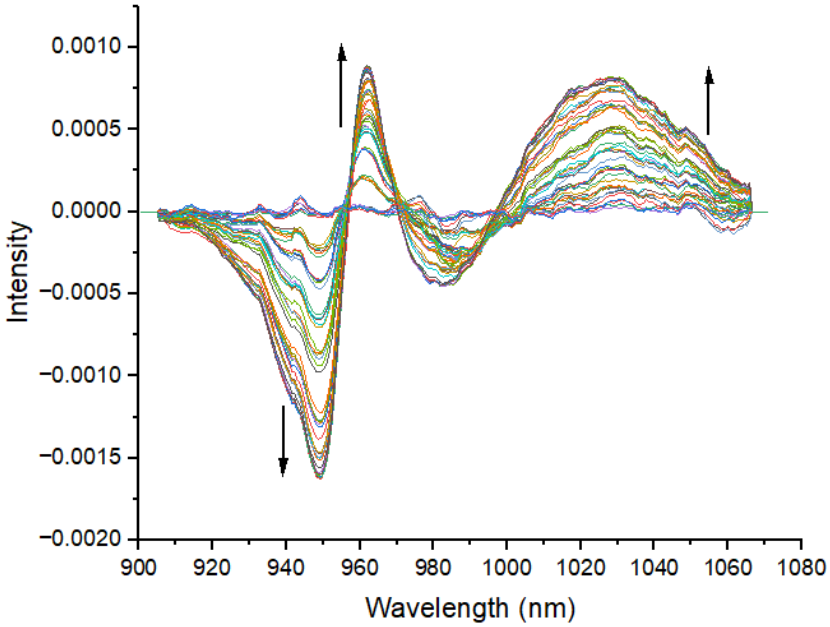

Figure S2. Near-infrared (NIR) water band region with calibration (12) and validation (40) spectra after applying a first derivative. Arrows indicate the direction of intensity increases with increasing HNO_3 concentration.

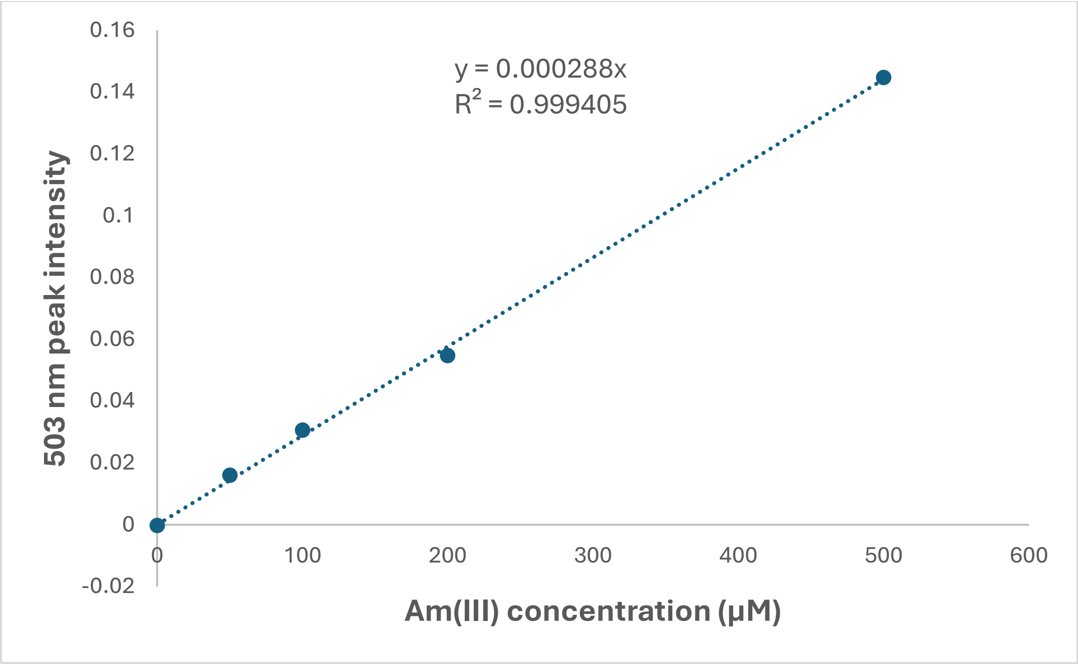

Figure S3. Linear regression of peak intensity vs. Am(III) concentration for samples collected at 4 M HNO_3.

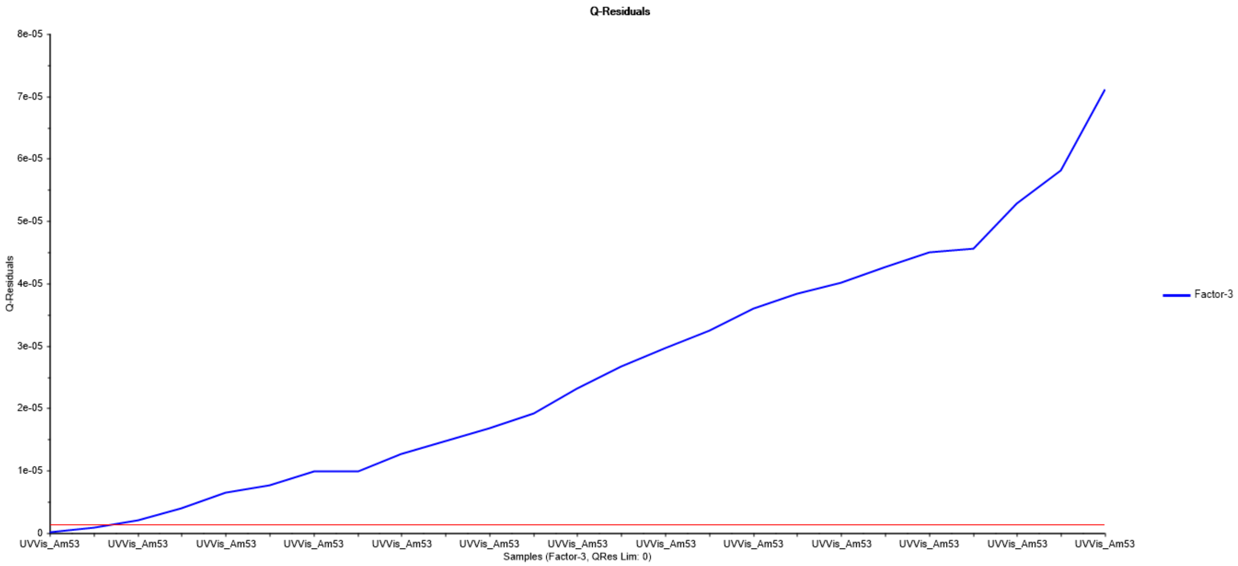

Figure S4. Q-residuals for predicted Am(III) samples while diluting with U(VI) using the PLS2 model. The red horizontal line represents the critical limit (1%).

Table S1. Gaussian–Lorentzian peak fit intensities for normalized peaks near 503 (1), 506 (2), and 511 nm (3). Peaks were fitted with OriginPro software.

HNO_3 (M)	Peak 1	Peak 2	Peak 3
0.1	0.7054	0.5117	0.0494
1	0.6941	0.5312	0.0612
2	0.6882	0.5469	0.0743
3	0.6801	0.5634	0.0875
4	0.6702	0.5335	0.1439
6	0.5989	0.4935	0.2924
7.5	0.5506	0.5507	0.3226
9	0.5303	0.5705	0.3449
